# Blast-Exposed Veterans With Mild Traumatic Brain Injury Show Greater Frontal Cortical Thinning and Poorer Executive Functioning

**DOI:** 10.3389/fneur.2018.00873

**Published:** 2018-11-09

**Authors:** Alexandra L. Clark, Victoria C. Merritt, Erin D. Bigler, Katherine J. Bangen, Madeleine Werhane, Scott F. Sorg, Mark W. Bondi, Dawn M. Schiehser, Lisa Delano-Wood

**Affiliations:** ^1^San Diego State University/University of California, San Diego (SDSU/UCSD) Joint Doctoral Program in Clinical Psychology, San Diego State University, University of California, San Diego, San Diego, CA, United States; ^2^VA San Diego Healthcare System, San Diego, CA, United States; ^3^Department of Psychology and the Neuroscience Center, Brigham and Young University, San Diego, CA, United States; ^4^Department of Psychiatry, School of Medicine, University of California, San Diego, San Diego, CA, United States; ^5^Center of Excellence for Stress and Mental Health, VA San Diego Healthcare System, San Diego, CA, United States

**Keywords:** cortical thickness, cortical thinning, mTBI, head injury, blast, blast TBI, blast exposure, subconcussive blast exposure

## Abstract

**Objective:** Blast exposure (BE) and mild traumatic brain injury (mTBI) have been independently linked to pathological brain changes. However, the combined effects of BE and mTBI on brain structure have yet to be characterized. Therefore, we investigated whether regional differences in cortical thickness exist between mTBI Veterans with and without BE while on deployment. We also examined whether cortical thickness (CT) and cognitive performance differed among mTBI Veterans with low vs. high levels of cumulative BE.

**Methods:** 80 Veterans with mTBI underwent neuroimaging and completed neuropsychological testing and self-report symptom rating scales. Analyses of covariance (ANCOVA) were used to compare blast-exposed Veterans (mTBI+BE, *n* = 51) to those without BE (mTBI-BE, *n* = 29) on CT of frontal and temporal a priori regions of interest (ROIs). Next, multiple regression analyses were used to examine whether CT and performance on an executive functions composite differed among mTBI Veterans with low (mTBI+BE Low, *n* = 22) vs. high (mTBI+BE High, *n* = 26) levels of cumulative BE.

**Results:** Adjusting for age, numer of TBIs, and PTSD symptoms, the mTBI+BE group showed significant cortical thinning in frontal regions (i.e., left orbitofrontal cortex [*p* = 0.045], left middle frontal gyrus [*p* = 0.023], and right inferior frontal gyrus [*p* = 0.034]) compared to the mTBI-BE group. No significant group differences in CT were observed for temporal regions (*p*'s > 0.05). Multiple regression analyses revealed a significant cumulative BE × CT interaction for the left orbitofrontal cortex (*p* = 0.001) and left middle frontal gyrus (*p* = 0.020); reduced CT was associated with worse cognitive performance in the mTBI+BE High group but not the mTBI+BE Low group.

**Conclusions:** Findings show that Veterans with mTBI and BE may be at risk for cortical thinning post-deployment. Moreover, our results demonstrate that reductions in CT are associated with worse executive functioning among Veterans with high levels of cumulative BE. Future longitudinal studies are needed to determine whether BE exacerbates mTBI-related cortical thinning or independently negatively influences gray matter structure.

## Introduction

The use of improvised and other explosive devices—such as rocket propelled grenades and mortar rounds—during the conflicts in Afghanistan and Iraq has led to a stark increase in the prevalence of combat-related blast exposure (BE) in the military population. Indeed, more than 60% of United States (U.S.) service members returning from the Middle East reported two or more BEs during their deployment (1). Similarly, among a convenience sample of Operation Enduring Freedom/Operation Iraqi Freedom/Operation New Dawn (OEF/OIF/OND) Veterans, nearly 80% reported at least one close range BE (within 100 m) while overseas (2). Such BE, combined with the improvement of combat protective gear and medical response methods, has led to unprecedented rates of certain non-lethal blast-related injuries within returning service members. Although musculoskeletal injuries, hearing loss, and vestibular dysfunction are common consequences of BE, of particular concern are the high rates at which BE results in mild traumatic brain injury (mTBI) among OEF/OIF/OND Veterans (3, 4).

The physical mechanisms of blast-related neurotrauma are complex and likely distinct from those mechanisms involved in pure blunt-force injuries. Conceptually, explosive detonation results in an over-pressurized shockwave—or primary blast wave—that transmits through the skull and directly interfaces with, displaces, or damages neural tissue (5, 6). This primary blast wave may also cause rapid physical displacement of blood from the abdominal area to the cranial vault, damaging the cerebrovasculature and blood brain barrier (7–9). Additionally, the percussive forces associated with BE may cause blunt-force injury by propelling debris into a soldier's skull, and/or causing the skull to make impact with other solid objects. Both blast and blunt forms of injury are thought to account for the acute clinical signs and symptoms of mTBI (i.e., loss of consciousness [LOC], alteration of consciousness [AOC], and posttraumatic amnesia [PTA]). Further, the quantification of BE itself is challenging given that the *intensity* of explosive forces resulting in mTBI is difficult to operationalize and characterize. While it has been established that high-pressure BE may cause extensive neural damage in humans (10), the majority of OEF/OIF/OND Veterans are exposed to significantly smaller thresholds of BE—from different proximities—which also originate from various types of explosives, highlighting the difficulty in characterizing the nature and extent of blast-related injury in this population.

During the past decade, considerable efforts have been placed on characterizing the pathophysiological consequences, or precise neural and white matter changes, associated with mTBI. Advanced neuroimaging techniques (i.e., diffusion tensor imaging [DTI], resting state functional [rsfMRI]) have revealed that a host of structural and functional brain changes occur in military service members with blast-related mTBI [for review see (11)]. These include macro- and microstructural white matter alterations, cortical thickness and volumetric reductions, as well as functional network and connectivity changes. Across the various neuroimaging findings among mTBI samples, frontal and temporal regions appear to be especially vulnerable, although widespread, diffuse damage has also been observed (11–13). Importantly, the nature of brain changes may fundamentally differ based on the manner in which the injury was sustained (e.g., blast/blunt force combination, blast only, blunt only), although the precise independent contributions of each mechanism are especially challenging to disentangle given that they frequently co-occur at the time of injury.

While BE may frequently result in an mTBI, low level—or subconcussive—BE may exert its own negative influence on the brain. For example, studies of OEF/OIF deployed service members have shown that BE, *independent* of diagnosis of mTBI, was associated with an increased likelihood of having decreased white matter microstructural integrity compared to controls (14, 15). These results were further corroborated by another study that examined serum markers of neuronal injury (e.g., ubiquitin C-terminal hydrolase-L1, αII-spectrin breakdown products, and glial fibrillary acidic protein) in members of the New Zealand Defense Force who did not experience an mTBI while participating in explosives training (16). Indeed, results revealed that (1) several participants showed increased levels of serum biomarkers of neuronal injury (i.e., ubiquitin C-terminal hydrolase-L1, αII-spectrin breakdown products) after low-level, subconcussive BE, and (2) higher levels of a serum biomarker composite were significantly associated with poorer performance on a neurocognitive composite. Similarly, (17) found that instructors, relative to students, endorsed more severe neurological symptoms, worse recognition memory, and fMRI differences after a 2-week period of subconcussive BE during a breacher basic training course; the authors attributed these differences to the fact that instructors, by nature of their profession likely have greater cumulative lifetime levels of blast exposure. Importantly, results from human studies align well with animal models with respect to neuropathological and neurobehavioral changes, although the subconcussive effects of BE can be difficult to operationalize and model in both human and animal studies. Nevertheless, experimental manipulation of peak pressure in mice and rats has revealed that even low levels induce neuronal loss, white matter alterations, cell signaling disruptions, and behavioral changes (18–20). Moreover, ultrastructural brain changes, poorer motor and memory performance, as well as increased anxiety levels, have been observed in mice exposed to primary low-intensity blast in the absence of head motion (21).

Recent research suggests that *cumulative* BE warrants careful consideration in Veteran samples, as several studies have revealed a dose-response relationship between BE, neurologic changes, and poor behavioral outcomes. For example, Ivanov et al. (22) recently found that a greater number of BEs (whether they resulted in a head injury or not) was significantly associated with reduced white matter microstructural integrity of the cingulum bundle. Similarly, using F-fluorodeoxyglucose (FDG) positron emission tomography (PET), greater cumulative BE was significantly associated with decreased neuronal activity in several regions of both the cerebrum and cerebellum of previously deployed military service members (23). Finally, greater cumulative BE has also been linked to negative behavioral outcomes, including poorer performance on verbal memory (24) and worse post-concussive symptom reporting (25). Combined, this literature not only suggests that BE itself may be detrimental to brain structure and function, but that individuals with greater cumulative levels of subconcussive BE may acquire more significant brain pathology, and thus be at an increased risk for worse clinical and functional outcomes.

Given that the combination of BE and mTBI may be especially deleterious to brain structure and cognition, we examined such effects by: (1) investigating regional gray matter morphological differences (i.e., cortical thickness) in vulnerable frontal and temporal regions in mTBI Veterans who were and were not exposed to blast (mTBI+BE vs. mTBI-BE); and (2) determining the relationship between cortical thickness and cognitive outcomes across different levels of BE. We hypothesized that BE would be associated with increased cortical thinning, and that reduced cortical thickness would be associated with worse cognitive performance in those with higher levels of cumulative BE. To our knowledge, this study represents the first to explore the neural and cognitive consequences of BE within the context of mTBI.

## Methods

### Participants and procedures

This sample included 80 OEF, OIF/OND Veterans with a history of mild TBI who were divided into those who were blast-exposed (*n* = 51, mTBI+BE) and those with no blast exposure (*n* = 29, mTBI-BE). Participants were recruited from posted paper and television advertisements located throughout the VA San Diego Healthcare System (VASDHS). Study procedures consisted of neuropsychological testing and the completion of a TBI clinical interview, self-report questionnaires, and magnetic resonance imaging (MRI) brain scans. Neuropsychological testing, clinical interviews, and questionnaire completion took place at the Veterans Medical Research Foundation located at the La Jolla VASDHS campus. MRI scans occurred at the University of California, San Diego (UCSD) Center for Functional MRI. This study was carried out in accordance with the recommendations of Institutional Review Boards (IRB) of the VASDHS and University of California, San Diego. The protocol was approved by VASDH and UCSD IRBs. All subjects gave written informed consent in accordance with the Declaration of Helsinki.

Diagnosis of mild TBI was based upon the guidelines detailed in the (26), which was defined as an LOC < 30 min, AOC up to 24 h, and/or PTA < 24 h. A “total number of lifetime TBIs” was created by summing the total number of injuries determined to have met VA/DoD criteria for mTBI for each participant. Additionally, a “most significant TBI” variable was created by directly comparing the presence and duration of LOC vs. AOC for each mTBI; injuries where an LOC was sustained were considered more severe than those with an AOC only. Finally, the “months since most recent TBI” was determined by calculating the difference between each participants' testing and their last reported mTBI.

The TBI clinical interview assessed head injuries sustained prior to, during, and following any military deployment. Under the direct supervision of a neuropsychologist (DS, LDW), trained graduate-level students and/or post-baccalaureate research assistants administered TBI history interviews. This interview allows for comprehensive assessment and staging of up to 10 lifetime brain injuries and was adapted from the VA Semi-Structured Clinical Interview for TBI (27). During the interview, each participant was queried about the context (e.g., military vs. non-military event) and mechanism (blast-related vs. blunt/mechanical force) of each reported head-injury. Since medical records pertaining to injuries sustained overseas and in combat settings are frequently not available or documented, we relied on retrospective self-report of critical information related to the presence and duration of any reported loss of consciousness (LOC), alteration of consciousness (AOC), and/or posttraumatic amnesia (PTA) to determine whether the injury met diagnostic criteria for mild TBI. However, patient's VA medical charts were reviewed for consistency of head injury reporting during our comprehensive clinical interview.

Participants were first queried about the number of times they were exposed to any blast detonation(s) that occurred within 100 meters (i.e., the distance of a professional football field) while on deployment. For each reported BE, details about the location, context (combat vs. non-combat), and direction (i.e., front, back, left, right) from which the BE was initiated were coded. Next, BE was categorized as a concussive (i.e., reported LOC, AOC, or PTA) or subconcussive (i.e., did not result in clinical symptoms of LOC, AOC, or PTA) injury. Participants with mTBI (due to blunt or blast-related mechanisms of injury) who also reported experiencing at least one *subconcussive* BE during their military service were considered to belong to the mTBI+BE group. mTBI participants who denied *any* exposure to blast while on deployment were considered to belong to the mTBI-BE group, and by nature of operationalization only had blunt mTBIs. Given that we were also interested in exploring the negative effects of *cumulative* BE on brain structure and function, the median number of subconcussive BEs that occurred within 100 meters was calculated and used to further divide the mTBI+BE group into those with low (*n* = 22, mTBI+BE Low) vs. high (*n* = 26, mTBI+BE High) levels of subconcussive BE. Importantly, these dichotomizations are sample specific, as cumulative levels of blast exposure may differ across other samples, branches, or professions within the military.

The following exclusion criteria were applied to the study sample: (1) history of any TBI that was classified as moderate (LOC > 30 min but < 24 h, AOC > 24 h, PTA > 1 day but < 7 days) or severe (LOC ≥ 24 h, AOC > 24 h, or PTA ≥ 7 days); (2) history of any neurological disorder (e.g., epilepsy, multiple sclerosis, stroke, chronic fatigue syndrome) other than TBI; (3) history of a major mental illness (e.g., schizophrenia, bipolar, or psychotic disorder) other than depression or post-traumatic stress disorder; (4) current substance/alcohol abuse as per *Diagnostic and Statistical Manual of Mental Disorders—Fourth Edition, Text Revision* (DSM-IV-TR) criteria (28); (5) current or prior history of substance/alcohol dependence as per DSM-IV-TR criteria; (6) a positive toxicology screen as measured by the Rapid Response 10-drug Test Panel; (7) any contraindications to MRI scanning (e.g., pregnancy, presence of metal); (8) any gross abnormalities, visible lesions, cortical contusions on T1 structural MRI scans, and (9) poor performance as defined by established cut-offs on the Test of Memory Malingering [TOMM; (29)] or Forced Choice Recognition trial of the California Verbal Learning Test-2nd Edition [CVLT-II; (30)].

### Self-report symptom rating scales

Participants completed self-report symptom rating scales. The PTSD Checklist (PCL-M) was used to capture current levels of posttraumatic stress (31). The Beck Depression Inventory-II (BDI-II) was used to capture current levels of depressive symptoms (32). The Neurobehavioral Symptom Inventory (NSI) was used to assess current levels of post-concussive symptoms (33).

### Executive functions factor

Participants were administered the following neuropsychological tests which emphasized executive functions, given that this cognitive domain is most commonly affected in mTBI samples: Trail Making and Verbal Fluency tests from the Delis-Kaplan Executive Function System [D-KEFS; (34)] and Wisconsin Card Sorting Test [WCST; (35)]. Additionally, the Reading subtest of the Wide Range Achievement Test 4 [WRAT4; (36)] was administered. Three mTBI+BE participants did not complete all of the neuropsychological testing and thus were excluded from secondary cognitive analyses. Raw scores were converted to demographically corrected standardized scores (e.g., scaled scores or T-scores) using accompanying normative data for the following neuropsychological variables: WCST Total Errors, WCST Perseverative Errors, DKEFS Verbal Fluency Switching Total Correct, DKEFS Verbal Fluency Accuracy, and DKEFS Number-Letter Switching. Next, the five variables were reduced into one executive functions factor using principal component analysis with Varimax rotation. The executive functions factor was determined to have acceptable internal consistency (α = 0.722).

### Neuroimaging data acquisition

Participants were scanned on a 3-Tesla General Electric MR750 system with an eight-channel head coil. A high-resolution T1 anatomical scan was acquired in the sagittal plane using a 3D FSPGR sequence with the following parameters: FOV = 24 cm, 256 × 192 matrix, *TR* = 8.1 ms, *TE* = 3.192 ms, flip angle = 12°, *TI* = 550 ms, bandwidth = 31.25 kHz, and 172 1.2 mm slices. After image acquisition, all T1 images underwent visual inspection for quality control purposes in an effort to ensure any artifacts that might affect image processing (e.g., motion, field inhomogeneity) were minimal.

### Neuroimaging processing

**Figure 1:**
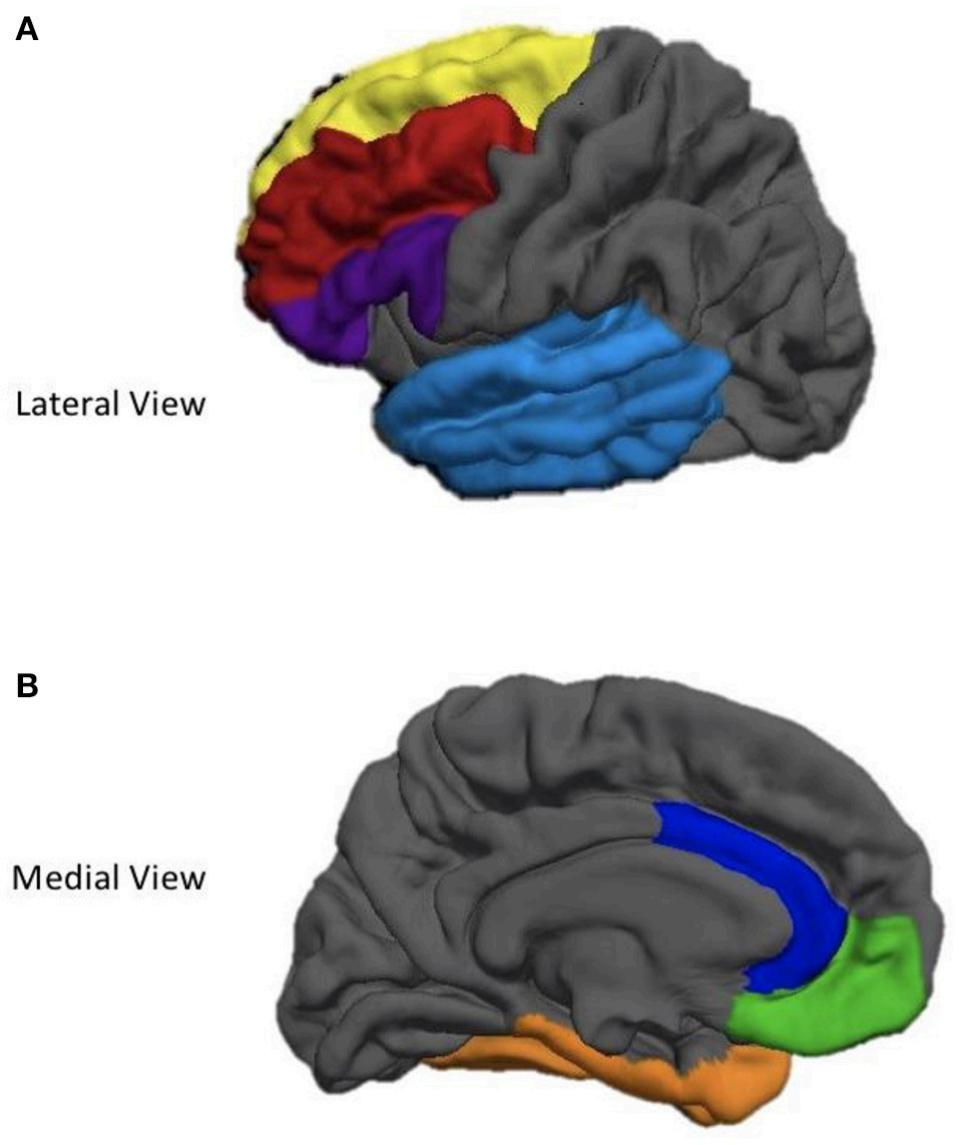
Depiction of cortical regions of interest utilized in the current study. **(A)** Yellow, Superior Frontal gyrus; Red, Middle Frontal Gyrus; Purple, Inferior Frontal Gyrus; Light Blue, Lateral Temporal Lobe. **(B)** Blue, Anterior Cingulate Cortex; Green, Oribitofrontal Cortex; Orange, Medial Temporal Lobe.

Cortical surfaces on all T1 images were reconstructed and parcellated into regions of interest (ROIs) using FreeSurfer 5.1 *recon-all* processing pipeline (37). FreeSurfer—a freely available cortical and subcortical segmentation and parcellation software suite—utilizes a series of automated imaging algorithms to (1) remove non-brain tissue, (2) conduct a Tailarach transformation, (3) segment cortical and subcortical white and gray matter structures, (4) perform nonparametric nonuniform intensity normalization of intensity values, (5) tesselate gray and white matter boundaries, (6) topology correct, (7) surface deform intensity gradients to optimally place gray/white and gray/CSF borders at the location where the greatest shift in intensity defines the transition to the other tissue class (38, 39). Next, data undergoes surface inflation and spherical registration to match individual cortical folding patters to expected cortical geometry across subjects. This produce a mesh of the pial and the white matter surfaces (38, 39). Cortical thickness was calculated as the measure of the distance (in millimeters) between the gray/white matter boundary to the gray matter/cerebral spinal fluid boundary at each vertex on the cortical surface. Importantly, cortical thinning is thought to represent trauma-induced synaptic pruning or apoptosis. FreeSurfer's measurement of cortical thickness has been validated using both manual (40) and histological analysis techniques (41). The Desikan-Killiany atlas was used to parcellate and label each hemisphere into 32 independent regions (42). The weighted average of several smaller ROIs [see (42) for these precise subdivisions] were used to obtain a mean cortical thickness value for each hemisphere in the following frontal and temporal lobe ROIs: (1) superior frontal gyrus (SFG), (2) middle frontal gyrus (MFG), (3) inferior frontal gyrus (IFG), (4) orbitofrontal cortex (OFC), (5) anterior cingulate cortex (ACC), (6) medial temporal lobe (MTL), and (7) lateral temporal lobe (LTL). See Figure 1 for a depiction of the ROIs utilized in this study.

### Statistical analyses

Analyses of variance (ANOVAs) were performed to determine whether the groups (mTBI+BE vs. mTBI-BE) differed on basic demographic variables, quantitative TBI injury characteristics, and self-report symptom rating scales. Chi-squared analyses were utilized to examine group differences on categorical demographic and TBI injury variables. Analyses of covariance (ANCOVAs) were used to determine whether the groups differed on cortical thickness ROIs. Regression analyses were used to determine whether cortical thickness was associated with cognition. All statistical analyses were conducted using the Statistical Package for the Social Sciences (SPSS) version 21 (SPSS IBM, New York, USA).

## Results

### Sample demographic and injury characteristics

The mTBI+BE group reported an average of 11.25 BEs (*Median* = 4, *Range* = 1–149). Participant demographics are presented in Table 1. The mTBI+BE group did not differ from the mTBI-BE group on age, ethnicity, education, or psychiatric symptomatology (all *p*-values > 0.05). However, the mTBI+BE group had a significantly higher proportion of men (*p* = 0.001), greater number of lifetime TBIs (*p* < 0.001), and more severe post-concussive symptoms (*p* < 0.02) relative to the mTBI-BE group. The mTBI+BE group also had a higher proportion of blast-related TBIs for their most significant injury (*p* < 0.001) and differed by branch of service (*p* < 0.001) relative to the mTBI-BE group.

**Table 1:** Sample characteristics, mean (SD).

	**Overall sample (*n* = 80)**	**mTBI+blast (*n* = 51)**	**mTBI-blast (*n* = 29)**	***F* or χ^2^**	***p***
Age	31.64 (7.28)	31.25 (7.45)	32.31 (7.05)	0.39	0.53
Education	13.83 (1.58)	13.80 (1.58)	13.86 (1.62)	0.03	0.88
WRAT-4 reading standard score Missing	101.59 (11.77) 2	102.10(11.53) 0	100.63 (12.43) 2	0.27	0.61
Sex (% Male)	89%	98%	72%	14.45	**0.001**^a^
**ETHNICITY**					
Caucasian African American Hispanic Asian Native American	50% 9% 30% 10% 1%	55% 4% 29% 10% 2%	42% 17% 31% 10% 0%	5.19	0.27^b^
**BRANCH OF SERVICE**					
Navy Army Marines Air Force	29% 28% 38% 6%	14% 35% 47% 4%	55% 14% 21% 10%	18.91	0.000^b^
PCL-M Total	46.96 (18.64)	49.73 (18.65)	42.11 (17.91)	3.17	0.08
BDI-II Total	21.64 (12.45)	21.66 (12.42)	21.59 (13.19)	0.000	0.98
NSI total Missing	36.08(17.85) 6	38.98 (17.25) 5	29.68 (17.23) 1	6.21	**0.02**
Total number of TBIs	2.61 (1.45)	3.06 (1.49)	1.83 (0.85)	10.97	**0.000**
% with single vs. multiple TBI history	23%, 77%	12%, 80%	41%, 59%	9.30	**0.002**^a^
Months since most recent TBI Missing	58.72 (42.03) 1	58.24 (43.42) 1	59.55 (40.23) 0	0.02	0.90
**MOST SIGNIFICANT INJURY**					
% LOC % AOC	62% 38%	61% 39%	65% 35%	0.18	0.67
**MOST SIGNIFICANT TBI TYPE**					
% Blast % Blunt % Blast with Secondary/Tertiary Blunt	23% 67% 10%	35% 49% 16%	0% 100% 0%	21.90	**0.000**^a^

*WRAT4, Wide Range Achievement Test 4; TBI, traumatic brain injury; LOC, loss of consciousness; AOC, alteration of consciousness; PTA, post-traumatic amnesia*.

a *Fischer's Exact Test*.

b *Likelihood Ratio*.

*Bold values indicate significant p-values*.

### Cortical thickness differences across mTBI+BE vs. mTBI-BE groups

A series of ANCOVAs were performed in order to determine whether the groups differed in cortical thickness of lateralized frontal ROIs. ANCOVAs controlling for age, sex, PCL-M total score, and total number of TBIs revealed a main effect of group such that the mTBI+BE group displayed significantly thinner cortices relative to the mTBI-BE group for the left MFG [*F*_(1, 74)_ = 5.38, *p* = 0.023, η_*p*_^2^ = 0.07], left OFC [*F*_(1, 74)_ = 4.17, *p* = 0.045, η_*p*_^2^ = 0.05], and right IFG [*F*_(1, 74)_ = 4.66, *p* = 0.034, η_*p*_^2^ = 0.07]. There was a trend toward significance with the mTBI+BE group displaying thinner cortices than the mTBI-BE group for the right SFG [*F*_(1, 74)_ = 3.65, *p* = 0.06, η_*p*_^2^ = 0.05]. Results revealed no significant differences in cortical thickness between the groups for the right and left ACC (*p's* >0.167), left IFG (*p* = 0.305), right OFC (*p* = 0.17), and right MFG (*p* = 0.23).

A second series of ANCOVAs were performed in order to determine whether the groups differed in cortical thickness of temporal ROIs. ANCOVAs controlling for age, sex, PCL-M total score, and total number of TBIs revealed there were no significant group differences for the lateralized MTL (*p's* > 0.237) and LTL (*p's* > 0.245).

### Cortical thickness and cognitive associations in the mTBI+BE group

A set of multiple linear regressions was performed in an effort to determine whether, independent of age and PTSD symptoms, there was a significant association between cortical thickness of the ROIs that differed between the mTBI+BE and TBI-BE groups and our executive functions factor. We chose to focus on brain-behavior relationships in the ROIs that significantly differed between the groups (i.e., left orbitofrontal cortex, left middle frontal gyrus, and right inferior frontal gyrus) in an effort to minimize multiple comparisons while better understanding the behavioral significance of the observed brain differences for all subsequent analyses. In each model, age, PCL-M total score, and a brain ROI were entered as independent variables, whereas the executive functions factor was the dependent variable. Results revealed no significant associations for any of the ROIs and our executive functions factor (all *p*-values > 0.07).

### Cortical thickness and cognitive associations by BE thresholds in the mTBI+BE group

**Figure 2:**
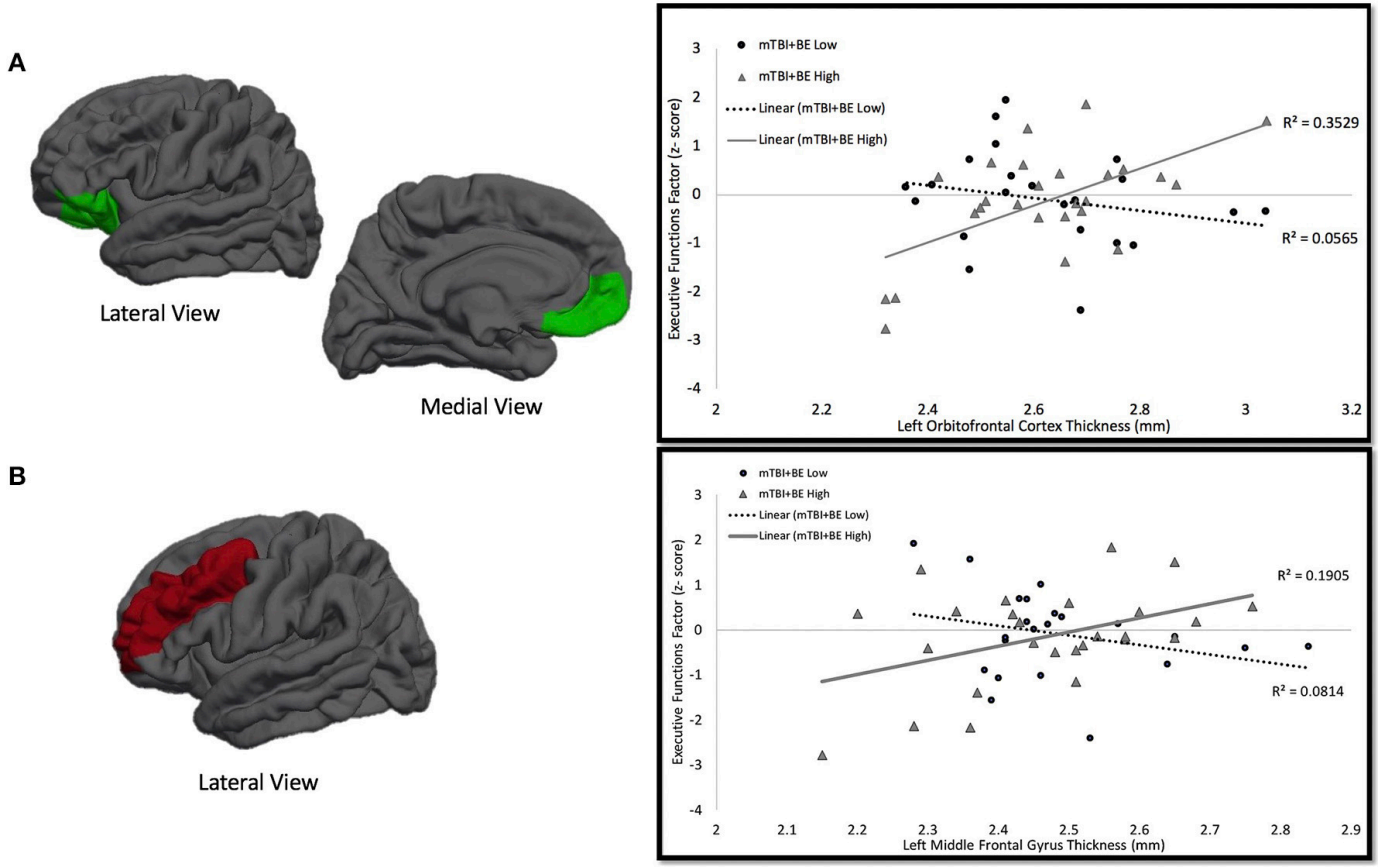
mTBI+BE Low vs. High × Cortical Thickness on Executive Functions Factor Interaction. **(A)** One the left side is a depicition of the left oribitofrontal cortex. On the right is the association between thickness of left orbitofrontal cortex and performance on an executive functions factor. The circles and dotted line represent the mTBI+BE Low group, whereas the triangles and solid line represent the mTBI+BE High group. **(B)** One the left side is a depicition of the left middle frontal gyrus. On the right is the association between thickness of left middle frontal gyrus and performance on an executive functions factor. The circles and dotted line represent the mTBI+BE Low group, whereas the triangles and solid line represent the mTBI+BE High group.

Secondary multiple regression analyses were performed in order to determine whether BE thresholds moderated the association between cortical thickness and performance on the executive functions factor. The mTBI+BE group was dichotomized into those with low (*n* = 22, mTBI+BE Low) and high (*n* = 26, mTBI+BE High) BE via a median split of the total number of self-reported blast exposures (*Median* = 4). Three subjects did not complete all neuropsychological testing and thus were excluded from subsequent analyses. For each model, the executive functions factor was entered at the dependent variable; independent variables included age, the PCL-M total score, BE grouping (mTBI+BE High vs. mTBI+BE Low), and cortical thickness of the relevant ROI. Results revealed a significant left OFC × blast exposure interaction on the executive functions factor (β = 6.39, *t* = 3.42, *p* = 0.001). Examination of simple main effects revealed that there was a significant positive correlation between cortical thickness of left OFC and the executive functions factor (*r* = 0.60, *p* = 0.001, *n* = 26) in the mTBI+BE High exposure group, but no such association in the mTBI+BE Low exposure group (*r* = −0.23, *p* = 0.29, *n* = 22). Similarly, a significant left MFG × blast exposure interaction on the executive function factor was observed (β = 5.67, *t* = 2.42, *p* = 0.02). Examination of simple main effects revealed that there was a significant positive correlation between cortical thickness of the left MFG and the executive functions factor (*r* = 0.43, *p* = 0.03, *n* = 26) in the mTBI+BE High exposure group, but no such association in the mTBI+BE Low exposure group (*r* = −0.29, *p* = 0.19, *n* = 22; see Table 2 and Figure 2). Finally, there was no significant right IFG x blast exposure interaction on the executive functions factor (β = −1.785, *t* = −9.23, *p* = 0.36).

**Table 2:** Multiple linear regression models for cortical thickness × blast exposure grouping (mTBI+BE high vs. mTBI+BE low) on executive functions factor.

	***R^2^***	***F***	**B**	**Std. error**	**β**	***t***	***p***
**LEFT ORBITOFRONTAL CORTEX**							
	0.43	6.38	–	–	–	–	<**0.001**
Age	–	–	−0.031	0.02	−2.28	−1.89	0.06
PCL-M total score	–	–	−0.022	0.01	−0.040	−3.41	0.001
Low vs. high blast grouping	–	–	−12.41	3.65	−6.25	−3.40	0.001
Left OFC cortical thickness	–	–	−5.74	2.20	−0.098	−2.61	0.01
Low vs. high blast grouping × left OFC cortical thickness	–	–	4.75	1.39	6.39	3.42	**0.001**
**LEFT MIDDLE FRONTAL GYRUS**							
	0.32	3.99	–	–	–	–	**0.005**
Age	–	–	−0.39	0.02	−0.028	−2.13	0.04
PCL-M total score	–	–	−0.019	0.01	−0.034	−2.66	0.01
Low vs. high blast grouping	–	–	−11.15	4.62	−5.61	−2.41	0.02
Left MFG cortical thickness	–	–	−6.56	3.14	−0.093	−2.09	0.04
Low vs. high blast grouping × left MFG cortical thickness	–	–	4.52	1.86	5.67	2.43	**0.02**

*Bold values indicate significant p-values*.

## Discussion

We explored whether BE was associated with reduced cortical thickness as well as the influence of cumulative BE on cognition in Veterans with history of mTBI. Results showed that, relative to those with mTBI who had not been exposed to blast while on deployment, Veterans with both BE and mTBI demonstrated significantly thinner cortices in frontal regions of the cerebrum. Moreover, in those with greater cumulative BE, reduced cortical thickness was significantly associated with poorer performance on tasks of executive function. These findings suggest that there is an association between cortical thinning and concomitant cognitive impairment post-deployment Veterans with mTBI and BE.

Although cortical thinning has previously been demonstrated within Veteran mTBI samples (43–45), no known studies have explored whether the *combination* of BE and mTBI negatively influences gray matter structure. As hypothesized, we found that those with both mTBI and BE showed greater frontal cortical thinning relative to those with mTBI alone. Results suggest that among Veterans with mTBI, BE —even at subconcussive levels—there may be an increased risk for negative brain changes. From a pathophysiological perspective, it remains unclear as to whether subconcussive BE exerts its own negative influence on gray matter structure or merely exacerbates mTBI-related cortical thinning. Findings from the animal literature have shown that BE of varying intensities (with associated head oscillation) results in neuronal loss (46) and the accumulation of tau, a protein associated with neurodegenerative diseases such as Alzheimer's disease or chronic traumatic encephalopathy in humans (47, 48). While speculative, we suspect that independent and interactive processes co-occur to produce poorer outcomes. Although there is considerable heterogeneity in both mTBI and blast-related injury, a synergistic effect may occur, particularly in overlapping areas of damage. There is a critical need for future, longitudinal studies to explore the precise mechanisms underlying gray matter changes due to BE in humans.

Previous behavioral and neuroimaging studies in Veterans have largely focused on characterizing the distinct effects of (1) blunt vs. blast-related mTBI, (2) subconcussive primary BE vs. pure blast mTBI (i.e., concussive injury due to primary BE without blunt injury), and/or (3) mTBI due to blunt or blast mechanisms vs. controls (15, 22, 49–52). While some of these studies have found cognitive, symptom, or neuroimaging differences across groups (15, 22, 51, 52), others have failed to find any categorical differences (15, 49, 50, 53). Our results suggest that failure to consider BE may explain—at least to some degree—the disparate findings observed across prior studies. Indeed, although findings of this study suggest that BE is an important factor influencing outcomes, few studies report or explore BE in their observed findings. This is especially important given that a recent study of military service members showed that approximately two-thirds of their mTBI sample reported BE while on deployment (4). Although no direct comparisons in symptom reporting between those with mTBI who were and were not BE were made, it is possible that the high prevalence of BE may have resulted in brain changes that contributed to the large proportion of individuals reporting postconcussive symptoms that persisted beyond the expected 3 month recovery window.

Interestingly, several recent studies have demonstrated that, independent of mTBI, close-range BE (i.e., within 10 m) is associated with both altered functional connectivity (54, 55) and verbal memory deficits in OEF/OIF/OND Veterans (24). That is, at least with respect to very close-range blast, *similar* blast-related brain and behavioral associations were observed in Veterans with or without mTBI. However, although proximity may be a critical factor with respect to the intensity and severity of blast-related neural injury, the close-range BE group within these studies reported a significantly greater number of BEs relative to the distance BE group (24, 54, 55). Thus, these findings may be reflective of cumulative BE, as opposed to (or in addition to) distance, as secondary analyses within one of these studies revealed that multiple distance BEs was also associated with reduced verbal memory performance (24). Finally, it is worthwhile to note that a greater proportion of Marines were represented in our mTBI+BE group, and others have shown that both Army and Marines service members are more likely to sustain close-range BE (24). Future research is needed to in order to (1) clarify how cumulative BE may differ as a function of occupation, combat, gender, training, or weaponry utilized and (2) quantify distinct thresholds of BE that may have negative brain or behavioral consequences in Veteran service members.

Results from our study also revealed that reduced cortical thickness was significantly associated with poorer performance on our executive functions factor in those with mTBI who had higher levels of cumulative BE, but not in those with mTBI who had lower levels of cumulative BE. While animal studies have shown that exposure to a single blast is sufficient to evoke neuronal loss and white matter degradation, recent work has shown that the severity of these brain changes are especially pronounced in mice with multiple BEs (23). Moreover, when compared to sham-exposed control mice, impaired motor performance was only observed in mice with multiple, as opposed to a single BE. It is possible that a certain threshold of neuronal damage must be reached before behavioral relationships are observed. A recent case study revealed that a Veteran with repeated BEs that never met clinical criteria for mTBI demonstrated significant white matter alterations, as well as impairments in processing speed, recognition memory, working memory, and executive function when compared to a reference control group (56). Critically, these results align with those of the present study in that they demonstrate that cumulative BE is associated with poorer behavioral outcomes—with greater numbers of BEs being more deleterious than a single blast.

The translation of animal studies of BE to explorations in humans has proven quite difficult and is not without serious limitations. Animal studies take place in controlled settings where distinct models of TBI (e.g., fluid percussive injury, controlled cortical impact, close head-injury) and/or types of explosives (e.g., live wire, shock tube, pressure generators) can be manipulated. Unfortunately, these factors are difficult to characterize in humans, as blast or blunt mechanisms of injury may independently or simultaneously occur, the precise quantification of blast intensity is virtually impossible given the combat setting, and preexisting vulnerabilities (e.g., substance use, individual differences in brain architecture and volume) may be at play.

One recent human neuropathological study tried to account for some of these factors by directly comparing brain specimens of male service members with chronic (*n* = 5) or acute/severe BE (*n* = 3) to civilians with impact, or blunt-related TBI (*n* = 5), prior exposure to opiates (*n* = 5), or no neurological conditions (57). Interestingly, both BE groups, which also consisted primarily of patients with antemortem PTSD diagnoses, demonstrated significantly greater astroglial scarring relative to the civilian groups. While intensity or severity of blast exposure could not be corroborated with objective data, the authors provide some evidence of distinct blast-related pathology relative to the other groups.

In another human neuropathological study (58), the authors compared brain specimens of Veterans with history of BE (*n* = 5) to several control groups—controls with history of opiate overdose (*n* = 6), controls with anoxic-ischemic encephalopathy (*n* = 6), controls with history of non-blast TBI (*n* = 5), and healthy controls (*n* = 7). The authors found evidence for increased amyloid precursor protein (APP)-positive axonopathy in blast-exposed Veterans relative to the control groups, providing additional support that BE results in distinct neuropathological patterns. Nevertheless, precise quantification of BE in humans is difficult, and other factors that predate military experience may also play a contributory role in our observed findings.

It is important to note that the current study has several limitations that warrant discussion. First, as is the case with most military TBI studies, we relied heavily upon retrospective self-report of both BE and head injury events that may have occurred many months or years prior; therefore, these events are subject to recall bias and could not be confirmed with medical documentation or field records at the time of injury. Similarly, while we conducted comprehensive TBI and BE interviews, many of the mTBI-BE group had been deployed to a combat zone and it is possible that some of members of the mTBI-BE group may failed to recall BEs that occurred within (or beyond) 100 m. Secondly, this was a cross-sectional research study, and results do not demonstrate that there have been changes in cortical thickness, only that thinner cortices were observed in Veterans with mTBI who were BE relative to those without BE. In other words, these are merely observed associations in one sample of Veterans and future longitudinal studies are needed to disentangle whether our observations represent changes or are merely the result of pre-injury differences in cortical thickness across the groups. There is evidence of neurological and psychiatric symptoms differences between BE and non-BE controls (59). Thus, additional comparison of BE and non-BE Veteran controls with no TBI history may help in clarifying the independent and/or synergistic effects of BE on mTBI, and we are currently collecting blast-related information for a subset of Veteran controls in order to clarify this possible relationship. Additionally, the mTBI+BE group was composed of mixed mechanisms of injury (i.e., blunt or blast-related mTBI) and had multiple TBIs relative to the mTBI-BE group. Although we controlled for the total number of TBIs in our analyses, it is difficult to disentangle the unique contributions of subconcussive blast, mTBI, and repetitive mTBI on cortical thinning in the present study. Moreover, although PTSD has also been linked to cortical thinning (60, 61) and cognition in Veteran mTBI samples (62), our mTBI+BE and mTBI-BE groups did not differ on this variable and we controlled for PTSD symptom severity in our analyses. Finally, additional work in this area should infuse other imaging metrics (e.g., arterial spin labeling) that may be more sensitive to blast-related brain changes, especially since mounting experimental animal evidence shows that blast-related head injury is associated with greater vascular pathology when compared to traditional blunt force TBI.

## Conclusion

This is the first known study to demonstrate that the combination of BE and mTBI (due to either blast or blunt force mechanisms of injury) negatively influences gray matter structure. Additionally, our results provide preliminary evidence that mTBI Veterans with both high levels of BE and reduced cortical thickness demonstrate reduced executive functioning, which is striking given that our sample is comprised of those with mild neurotrauma who are, on average, many years removed from their head injury event. Taken together, these findings suggest that Veterans with both mTBI and exposure to higher levels of blast may be at increased risk for both cerebral and behavioral changes post-deployment. Future prospective studies are needed to disentangle (1) the precise pathophysiological mechanisms underlying cortical thickness changes associated with BE, mTBI, and these comorbid conditions, and (2) the extent to which outcomes may differ based on distance, intensity, or severity of BE, and (3) the negative consequences of repetitive mTBI as opposed to repetitive subconcussive BE.

## Author contributions

AC and LD-W contributed to manuscript conception. AC performed some of the data collection, processed the neuroimaging data, and conducted all the statistical analyses. VM assisted with analyses and interpretation of results. AC drafted the paper with contributions from VM Paper comments and revisions were provided by MW, EB, KB, SS, MB, DS, and LD-W.

### Conflict of interest statement

The authors declare that the research was conducted in the absence of any commercial or financial relationships that could be construed as a potential conflict of interest.

## Abbreviations

ACC: anterior cingulate cortex
ANOVA: analysis of variance
ANCOVA: Analysis of covariance
AOC: alteration of consciousness
BDI-II: Beck Depression Inventory-II
BE: blast exposure
CT: cortical thickness
DKEFS: Delis-Kaplan Executive Function System
DTI: diffusion tensor imaging
IFG: inferior frontal gyrus
LOC: loss of consciousness
LTL: lateral temporal lobe
MFG: middle frontal gyrus
mTBI: mild traumatic brain injury
ROIs: regions of interest
MTL: medial temporal lobe
mTBI+BE: mTBI Veterans who were exposed to blast
mTBI-BE: mTBI Veterans were not exposed to blast
mTBI+BE Low: Veterans with mild traumatic brain injury with low blast exposure
mTBI+BE High: Veterans with mild traumatic brain injury with high blast exposure
MRI: magnetic resonance imaging
NSI: Neurobehavioral Symptom Inventory
OEF/OIF/OND: Operation Enduring Freedom/Operation Iraqi Freedom/Operation New Dawn
OFC: orbitofrontal cortex
PCL-M: PTSD Checklist-Military
PTA: posttraumatic amnesia
PTSD: posttraumatic stress disorder
rsfMRI: resting state functional
SFG: superior frontal gyrus
WCST: Wisconsin Card Sorting Test
WRAT-4: Wide Range Achievement Test 4.
